# Metabolomics fingerprinting of thyroid malignancies: a GC/MS-based approach for subtype classification and biomarker discovery

**DOI:** 10.1186/s12885-025-15073-0

**Published:** 2025-10-15

**Authors:** Raziyeh Abooshahab, Maryam Zarkesh, Mehdi Hedayati

**Affiliations:** 1 Cellular and Molecular Endocrine Research Center, Research Institute for Endocrine Molecular Biology, Research Institute for Endocrine Sciences, Shahid Beheshti University of Medical Sciences, PO Box: 19395-4763, Tehran, Iran; 2 Curtin Medical School, Curtin University, Bentley, 6102 Australia; 3 Curtin Health Innovation Research Institute, Bentley, 6102 Australia

**Keywords:** GC/MS, Metabolomics, Cancer metabolism, Metabolic profile, Thyroid cancers

## Abstract

**Background:**

Thyroid cancer encompasses distinct histological subtypes, each potentially associated with unique metabolic characteristics. However, the comprehensive metabolic reprogramming underlying these malignancies remains insufficiently characterized. Hence, this study aimed to identify untargeted metabolomics alterations in plasma samples from patients diagnosed with papillary thyroid carcinoma (PTC), follicular thyroid carcinoma (FTC), medullary thyroid carcinoma (MTC), and healthy controls, to elucidate potential metabolic signatures associated with each cancer type.

**Methods:**

Plasma samples from patients with PTC (*n* = 14), FTC (*n* = 8), and MTC (*n* = 15), along with samples from healthy subjects (*n* = 15), were collected for untargeted metabolomics analysis using gas chromatography-mass spectrometry (GC/MS). Multivariate and univariate analyses were performed for diagnostic assessment using MetaboAnalyst, SIMCA software, and R packages.

**Results:**

A total of 61 metabolites were annotated across all plasma samples. Multivariate analyses, including partial least squares discriminant analysis (PLS-DA) and orthogonal PLS-DA (OPLS-DA), revealed distinct group separations and demonstrated robust model performance. One-way ANOVA followed by Tukey’s HSD and variable importance in projection (VIP ≥ 1) highlighted 35 significantly altered metabolites. Among these, linolenic acid (*q* = 4.76E-13) and arachidonic acid (*q* = 1.39E-12) showed substantial reductions across all thyroid cancer subtypes. Conversely, glutamine (*q* = 1.14E-10), methionine (*q *= 2.54E-09), and 2-hydroxybutanoic acid (*q* = 1.49E-07) were elevated in FTC and PTC. A Random Forest analysis further highlighted linolenic, linoleic, arachidonic acids, methionine, glutamine, and pyruvic acid, as crucial discriminative elements, achieving a macro-averaged AUC of 0.956 in multi-class classification.

**Conclusion:**

This plasma metabolomics study reveals distinctive metabolic signatures associated with different thyroid cancer subtypes, suggesting potential biomarkers for differential diagnosis. These findings underscore the importance of metabolomics in enhancing subtype differentiation and provide insight into metabolic pathways associated with disease progression.

**Supplementary Information:**

The online version contains supplementary material available at 10.1186/s12885-025-15073-0.

## Introduction

Thyroid cancer (TC) is a rare malignancy in adults, accounting for less than 1% of all solid tumors [1]. Histologically, TC is categorized into three primary subtypes, each possessing distinct molecular and pathological characteristics that influence clinical behaviour and prognosis [2]. Differentiated thyroid cancer (DTC) is the most common subtype, representing approximately 90% of all thyroid tumors, and originates from thyroid follicular epithelial cells [3]. Medullary thyroid cancer (MTC), which arises from parafollicular C-cells, makes up about 10% of thyroid tumors [4]. Anaplastic thyroid cancer (ATC), a rare and highly aggressive form, constitutes roughly 1% of thyroid tumors and derives from follicular cells [5]. Cytological evaluation of fine-needle aspiration biopsy (FNAB) is widely accepted as a standard diagnostic procedure for thyroid cancer [6]. However, pathologists cannot definitively determine malignancy in 10–30% of cases, resulting in an indeterminate diagnosis [7]. Consequently, patients with suspicious nodules often undergo lobectomy or thyroidectomy for explicit histopathological assessment [7]. This approach can lead to overtreatment in instances where the lesion is ultimately benign [7, 8]. To enhance diagnostic accuracy and minimize unnecessary surgical interventions, researchers are actively exploring reliable biological markers that can assist in detecting and classifying indeterminate thyroid lesions, enabling more precise treatment decisions. Various molecular and cellular assays have been integrated into FNAB cytology to better identify indeterminate lesions [6]. Despite these advancements in diagnostics and therapeutics, challenges persist in the effective diagnosis and treatment of thyroid cancer. As a result, ongoing research aims to identify novel diagnostic and prognostic biomarkers to refine early detection, enhance risk stratification, and uncover potential therapeutic targets for thyroid cancers.

Metabolic reprogramming has emerged as a hallmark of cancer, reflecting profound alterations in cellular metabolism that support tumor initiation, growth, and survival [9]. Metabolomics, which involves thoroughly examining metabolites within biological systems, serves as an effective method for defining metabolic profiles specific to diseases and pinpointing possible biomarkers [6]. Previous studies have utilized metabolomics techniques, such as nuclear magnetic resonance (NMR) spectroscopy and mass spectrometry (MS), to study metabolic changes in thyroid cancer, with a primary focus on individual histological subtypes [6, 10, 11]. Recent work underscores the feasibility of plasma metabolomics for thyroid disease stratification. GC/MS plasma studies have differentiated thyroid nodules from healthy controls and mapped alterations across carbohydrate, amino acid, tricarboxylic acid (TCA) cycle, and lipid metabolism, highlighting the diagnostic promise of circulating metabolites [12]. Complementary NMR-based studies in papillary thyroid carcinoma (PTC) similarly report systemic metabolic reprogramming detectable in biofluids and tissue [13]. These investigations have revealed disrupted metabolic pathways related to energy metabolism, amino acid turnover, and lipid homeostasis, highlighting the potential of metabolomics in thyroid cancer studies. Beyond thyroid cancer, machine learning–assisted pipelines are increasingly used to prioritize discriminative metabolites and to benchmark classification performance in cancer metabolomics [14].

Despite progress, no thorough studies have systematically examined the metabolic profiles of PTC, FTC, and MTC to identify unique metabolic signatures for each subtype. Such analyses are crucial for revealing specific metabolic changes. In this study, we employed GC/MS-based metabolomics to investigate the metabolic alterations between PTC, FTC, and MTC and healthy individuals. By analyzing plasma samples from patients with these thyroid cancer types alongside healthy controls, we aimed to identify unique metabolic signatures that could enhance our understanding of the pathophysiology of these cancers and potentially serve as diagnostic or differential biomarkers.

## Materials and methods

### Study design

This cross-sectional study involved patients referred to the Cellular and Molecular Endocrine Research Center at the Research Institute for Endocrine Sciences, Shahid Beheshti University of Medical Sciences, and Shariati Hospital in Tehran, Iran, for near-total or total thyroidectomy from 2018 to 2019. Participants were selected based on the final surgical pathology reports, including only those with histopathological diagnoses of PTC, FTC, and MTC at the outset of the research. Blood samples were collected from the case group before surgery, which consisted of 14 patients with PTC (8 females and 6 males), 15 with MTC (10 females and 5 males), and 8 with FTC (5 females and 3 males). The diagnosis of thyroid malignancies was confirmed through pathological findings and clinical outcomes. Additionally, 15 healthy individuals (10 females and 5 males) were recruited as a control group at the same clinical centers. Each participant and healthy volunteer gave written informed consent. Participants using medications that affect thyroid function or those with other types of cancer and metabolic diseases, including metabolic syndrome, diabetes, and insulin resistance (IR), were excluded.

About 5 ml of blood was drawn in tubes containing anticoagulant ethylenediaminetetraacetic acid (EDTA). Before metabolomics analysis, plasma was separated immediately through blood centrifuging and kept frozen at − 80°C. The current study adhered to the principles outlined in the Declaration of Helsinki (1975) and was approved by the local Ethics Committee of Shahid Beheshti University of Medical Sciences, Tehran, Iran (IR.SBMU.ENDOCRINE.REC.1402.122).

### Plasma sample preparation for GC/MS analysis

Plasma metabolites were extracted and derivatized using our previously reported method with a minor modification [1]. In brief, 50 µL of plasma from each case was extracted with 1 mL of protein precipitant (methanol/water/isopropanol, 5:2:2, v/v/v). The tubes of mixtures were vortexed for 60 s and then chilled at − 20°C for 20 min, followed by centrifugation at 14,000 rpm for 15 min at 4°C. Then, the clear supernatant of each sample was collected and evaporated to dryness using an Eppendorf vacuum centrifuge, operated at 45°C for 4 h. Before injection, all dried samples underwent derivatization through a two-step process involving methoximation and trimethylsilylation (TMS). During the methoximation step, 40 µL of methoxyamine hydrochloride (20 mg/mL in pyridine) was added to the dried samples, then mixed for 30 s period. The mixtures were then incubated on a thermo-shaker at 900 rpm for 1 h at 60°C. Afterward, 60 µL of N-Methyl-N-(trimethylsilyl) trifluoroacetamide (MSTFA) was added as the silylating agent. The samples were then remixed and placed on the thermo-shaker to react at 900 rpm for 20 min at 45°C.

To generate a representative quality control (QC) sample, 15 µL aliquots from each resuspended plasma sample were pooled. QC samples were incorporated into the analytical sequence at regular intervals, specifically following every ten study samples, to assess instrumental stability and relative standard deviation (RSD%). The order of sample extraction and subsequent analysis was randomized to minimize potential biases associated with batch effects, instrumental drift, and/or underlying biological variation.

### GC/MS analysis

Gas chromatography–quadrupole mass spectrometry (GC/MS) analysis was performed using an Agilent 5975 C MSD coupled with an Agilent 7890 A GC system, which was equipped with an HP-5 ms capillary column (Agilent J&W, 30 m × 0.25 μm × 0.25 mm). A volume of 1 µL from each derivatized extract sample was injected at a split ratio of 4:1, using helium as the carrier gas at a flow rate of 1 mL/min. The inlet, MS transfer line, and quadrupole temperatures were maintained at 280, 230, and 150°C, respectively. All samples were processed in a randomized order. The initial oven temperature was sustained at 60°C for 1 min, then ramped at a rate of 10°C/min to a final temperature of 280°C with a hold time of 10 min. A post-run duration of 5 min was allowed for the oven to cool down to 60°C. The electron ionization (EI) source was set to 70 eV. GC/MS data acquisition was conducted using full-scan spectra (50–600 m/z), recorded in 37.5 min following a solvent delay of 5.4 min.

### Raw GC/MS data processing

ChemStation Data Analysis software (Agilent Technologies, Palo Alto, CA, USA) was employed to convert raw data into CDF format (NetCDF), which was then transformed into “abf” format using the Reifycs Abf converter. Automatic processing and analysis of deconvolution, peak identification, gap filling, and peak alignment of metabolites were conducted using MS-DIAL software, which features integrated MS/MS reference libraries (version 4.60). The original dataset included average retention time (RT), mass-to-charge ratio (m/z), InChIKey, and peak intensity metrics, which were exported from MS-DIAL for further analysis. Additionally, the NIST Mass Spectral Search Program (version 2.0) validated all metabolite spectra identified in MS-DIAL against reference spectra from the replib, mainlib, and Fiehn libraries, maintaining a similarity index of ≥ 70%.

### Statistical analysis

Statistical analyses involved both multivariate and univariate techniques. Probabilistic quotient normalization (PQN), log transformation, and mean centring were applied to all selected metabolites using MetaboAnalyst (v6.0, http://www.metaboanalyst.ca). SIMCA-P 14.0 software (Umetrics, Umeå, Sweden) was used to generate multivariate statistical plots, including principal component analysis (PCA), partial least squares discriminant analysis (PLS-DA), and orthogonal partial least squares discriminant analysis (OPLS-DA), to identify outliers and assess the degree of differences between experimental groups. Cross-validated predictive residuals (CV-ANOVA) and permutation tests were conducted to evaluate the reliability of the models. Variable importance in projection (VIP) scores equal to or greater than one was deemed important for model discrimination. The *p*-values were corrected using false discovery rate (FDR) calculations to address multiple testing issues. ‌Boxplots were created using the R packages “ggplot2”, “ggpubr”, and “tidyverse”. Correlation coefficients were calculated to explore relationships among significant metabolites and clinical as well as pathological characteristics, represented as a heatmap through the “corrplot” R package. Finally, metabolic pathway analysis was conducted using enrichment pathway analysis in MetaboAnalyst (v6.0).

### Machine learning analysis

Random Forest (RF) classification framework with nested cross-validation (nested CV) was implemented to identify discriminative metabolites that differentiate thyroid cancer subtypes from healthy controls. This approach was specifically designed to prevent information leakage and provide an unbiased estimate of model performance. For each outer fold, inner resampling was applied to optimize the mtry parameter, and out-of-fold (OOF) predictions from all outer loops were aggregated for final evaluation. The ROC curve for each class was computed using pROC, comparing each class against all others, and plotted in a multi-class ROC curve using the R package “ggplot2”. The macro-averaged AUC was then derived as the mean of individual class AUCs. Performance was further visualized through a normalized confusion matrix, highlighting accurate class separation. Feature importance was assessed on the final RF model trained on the full dataset, using two complementary criteria: Mean Decrease Accuracy (MDA) and Mean Decrease Gini (MDG). Finally, the statistical validity of the RF model was assessed through a permutation test with 100 iterations, in which sample labels were randomly shuffled.

## Results

### Clinical characteristics of the subjects

The clinical and pathological characteristics of the study participants are summarized in Table 1. A total of 52 individuals were included and categorized into four groups: PTC (*n* = 14), FTC (*n* = 8), MTC (*n* = 15), and healthy controls (*n* = 15). As shown in Table 1, the mean ages differed significantly among the PTC (33.14 ± 10.3), FTC (57.2 ± 16.6), MTC (47.07 ± 15.3), and healthy groups (34.6 ± 11.1) years (*P* < 0.001). The mean of tumor size (Cm) was significantly variable among PTC (1.61 ± 0.94), FTC (3.43 ± 1.72), and MTC (2.62 ± 1.21) patients (*P* = 0.009).

**Table 1:** Demographic and clinical-pathological features of patients

Variables		PTC (*n* = 14)	FTC (*n* = 8)	MTC (*n* = 15)	Healthy (*n* = 15)	^a^ *p*-value
Age (years)		33.1 ± 10.3	57.2 ± 16.6	47.1 ± 15.3	34.6 ± 11.1	< 0.001
Sex (%)	Male	6(42.9)	3(37.5)	5(33.3)	5(33.3)	0.945
	Female	8(57.1)	5(62.5)	10(66.7)	10(66.7)	
Tumor size (Cm)		1.61 ± 0.94	3.43 ± 1.72	2.62 ± 1.21	-	0.009
^b^ TNM stage based on AJCC	I&II	12(85.7)	4(57.1)	-	-	0.182
	III&IV	2(14.3)	3(42.9)	-	-	
Lymph node metastasis positive		8(57.1)	1(12.5)	6(40.0)	-	0.122

*PTC* Papillary thyroid cancer, *FTC* Follicular thyroid cancer, *MTC* Medullary thyroid cancer

^a^*p*-value < 0.05 was considered statistically significant

^b^American Joint Committee on Cancer (AJCC) Tumor-Node-Metastasis (TNM) staging system

### Untargeted metabolomics profiles of plasma samples among four groups

Data processing via MS-DIAL identified 568 GC/MS peaks, out of which 61 metabolites were structurally annotated and consistently reliable across all four groups: healthy, PTC, FTC, and MTC.

**Fig. 1:**
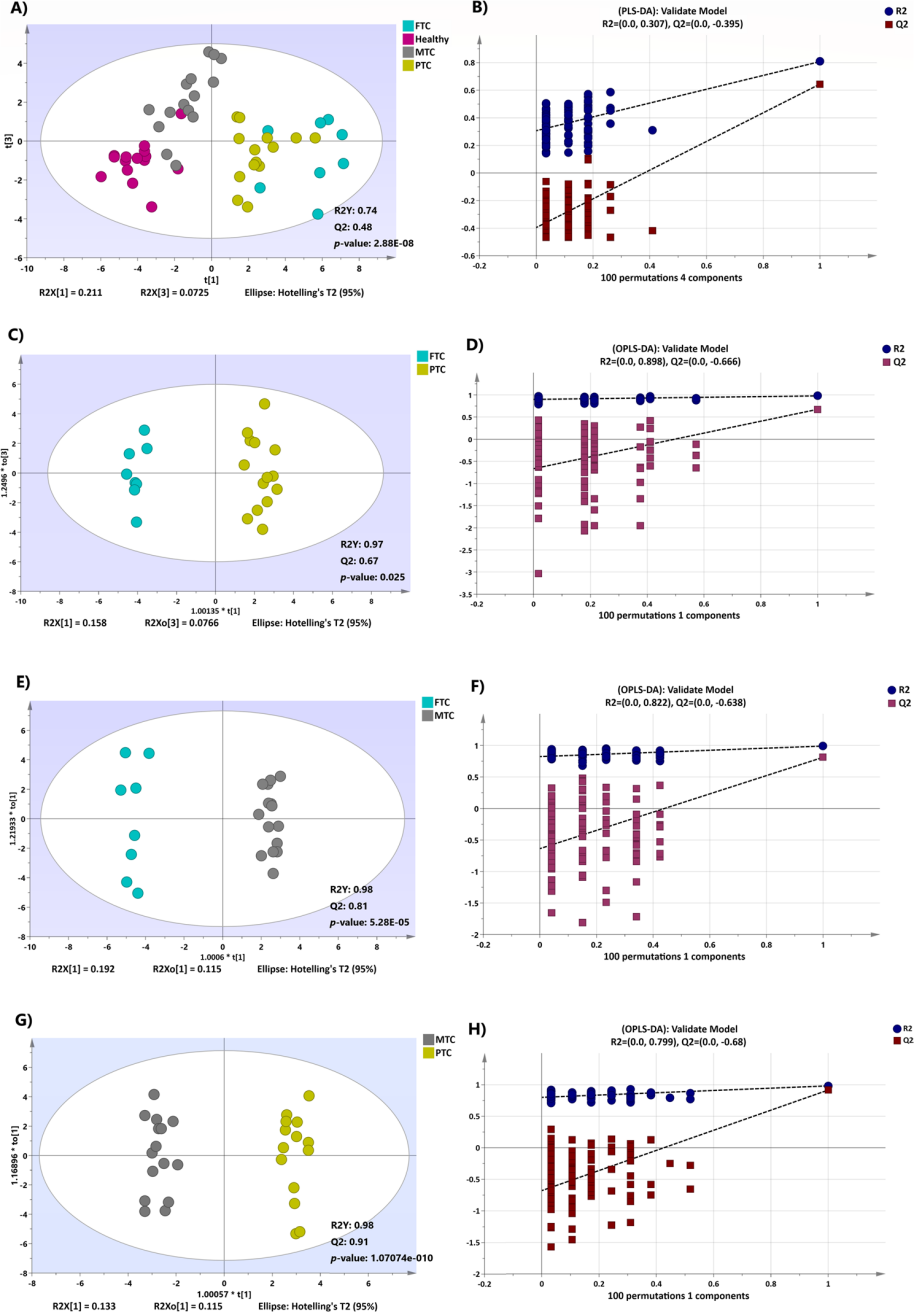
**A** Supervised PLS-DA analysis score scatter plots illustrate the distinction between the metabolic profiles of different groups. **B** The permutation test confirmed the significance of the model. **C**,** E**,** G** OPLS-DA analysis score scatter plots demonstrate that the metabolic profile of FTC is distinct from PTC **C**, FTC is distinct from MTC **E**, and MTC is distinct from PTC **G**. **D**,** F**,** H** Permutation tests with 100 permutations of the OPLS-DA models indicated that the models were significant

The PCA was performed to explore the variance structure and potential clustering among the samples. The resulting score plot (Fig. 1S) displays the distribution of samples along the first two principal components, which together explain 30.1% of the total variance. The QC samples form a tight cluster, indicating good analytical stability and method reproducibility. The 95% Hotelling’s T² ellipse shows that most samples fall within the expected confidence limits, with no significant outliers observed.

A supervised PLS-DA model was employed to identify metabolites contributing to group differentiation. The PLS-DA score plot demonstrated excellent clustering among the four groups, yielding acceptable predicted variance (R2Y score = 0.74) and predictive ability (Q2 score = 0.48). This indicates significant alterations in plasma metabolites associated with thyroid malignancies compared to healthy individuals (Fig. 1A). The CV-ANOVA test confirmed the model’s significance (*p*-value = 2.88E-08). Cross-validation through a permutation test (*n* = 100) indicated that the original model’s R2 and Q2 values outperformed the permuted model, demonstrating strong prediction capability (Fig. 1B). OPLS-DA plots were created to maximize separation between the malignancy groups: FTC vs. PTC, FTC vs. MTC, and PTC vs. MTC. As displayed in Figs. 1C–H, distinct separations were achieved in the score plots for each model, with satisfactory R2Y and Q2 values. Cross-validation through permutation tests (*n* = 100) confirmed that the original models maintained better R2 and Q2 values than the permuted models, indicating robust predictive performance.

**Fig. 2:**
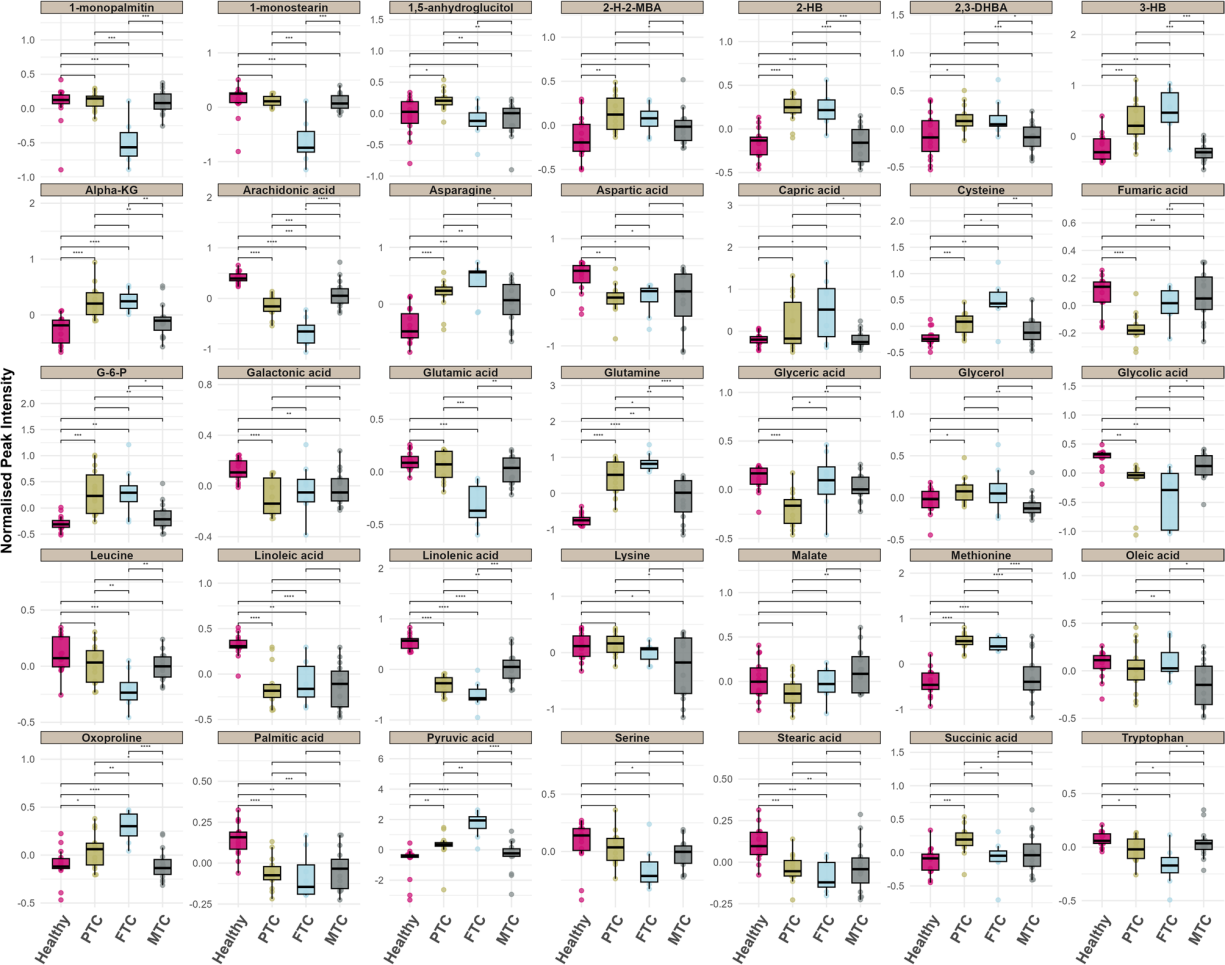
The boxplots depict the distribution of 35 metabolites with the highest significance (*p*-values < 0.05 and VIP scores ≥ 1) in the variance analysis. These plots enable comparisons among the four groups. On the x-axis, individual metabolites are represented for each group, while the y-axis indicates the normalized peak intensity. Significant differences in metabolites were determined using Tukey’s Honestly Significant Difference (TukeyHSD) test, denoted as (*) *p* ≤ 0.05, (**) *p* ≤ 0.01, and (***) *p* ≤ 0.001. The group key is as follows: PTC, papillary thyroid cancer; FTC, follicular thyroid cancer; MTC, Medullary thyroid cancer; 2-HB, 2-hydroxybutanoic acid; 3-HB, 3-hydroxybutyric acid; Alpha-KG, alpha-ketoglutaric acid; G-6-P, glucose-6-phosphate; 2,3-DHBA, 2,3-dihydroxybutanoic acid; 2-H-2MBA, 2-hydroxy-2-methylbutanoic acid

Furthermore, one-way ANOVA (*p*-values < 0.05 and *q*-values < 0.05) was conducted, along with Tukey’s HSD test and multivariate analysis, using a VIP score cutoff of ≥ 1, to identify metabolites that were significantly altered among the four classifications (Table 2). Our findings indicated that 35 out of the 61 metabolites varied significantly across these groups. The Boxplots in Fig. 2 illustrate the distribution of normalized peak intensities for significant metabolites among the four groups. Notably, linolenic acid and arachidonic acid displayed highly substantial reductions in the thyroid cancer subtypes compared to healthy controls (*q*-values = 4.76E-13 and 1.39E-12, respectively). Glutamine levels were significantly elevated in FTC and PTC compared to the healthy and MTC groups (*q*-value = 1.14E-10), indicating pronounced metabolic changes associated with FTC and PTC. Further, 2-hydroxybutanoic acid (2-HB) and methionine were elevated in FTC and PTC compared to healthy and MTC samples (*q*-values = 1.49E-07 and 2.54E-09, respectively). Additionally, metabolites such as linoleic acid, 1-monostearin, and glutamic acid showed significant decreases across cancer groups. 3-hydroxybutyric acid (3-HB) was increased in the PTC and FTC groups, while it was decreased in the MTC groups. Other metabolites exhibited substantial alterations, including oxoproline, fumaric acid, glyceric acid, and palmitic acid. Specifically, oxoproline levels increased in the cancer groups (*q*-value = 3.66E-05), whereas fumaric acid displayed mixed trends, with noticeable distinctions from healthy controls (*q*-value = 0.00016).

**Table 2:** Substantially altered metabolites among groups using One-way ANOVA analysis

Metabolites	VIP	One-way ANOVA		Multiple Comparisons Tukey HSD (^a^*p*-value)					
		^a^*p*-value	FDR	H/PTC	H/FTC	H/MTC	PTC/FTC	PTC/MTC	MTC/FTC
Linolenic acid	1.39	7.80E-15	4.76E-13	6.05E-14	1.57E-06	9.46E-06	0.143	0.0002	0.0003
Arachidonic acid	1.32	4.56E-14	1.39E-12	1.02E-08	4.54E-06	0.0004	0.0007	0.013	2.23E-05
Glutamine	1.25	5.60E-12	1.14E-10	1.29E-07	1.12E-08	0.001	0.014	0.003	6.21E-06
Methionine	1.42	1.66E-10	9.37E-09	1.53E-10	3.25E-08	0.3636	0.676	3.58E-05	6.61E-05
1-monostearin	1.65	8.21E-09	1.00E-07	0.921	0.0005	0.794	0.0009	0.761	0.0009
2-HB	1.30	1.46E-08	1.49E-07	5.94E-07	0.0003	0.972	0.929	3.71E-06	0.0003
Glutamic acid	1.44	4.75E-08	4.14E-07	0.319	0.0003	0.059	0.0005	0.447	0.001
3-HB	1.25	1.16E-07	8.88E-07	0.0005	0.0005	0.559	0.168	0.0001	0.0004
Linoleic acid	1.53	3.13E-07	3.61E-07	1.31E-06	0.001	4.34E-06	0.439	0.740	0.621
1-monopalmitine	1.58	6.48E-07	3.95E-06	0.572	0.0009	0.794	0.0006	0.666	0.0007
Cysteine	1.20	1.27E-06	7.05E-06	0.0009	0.001	0.144	0.025	0.100	0.005
Aspargine	1.13	2.68E-06	1.36E-05	1.37E-05	0.0001	0.002	0.129	0.230	0.026
Pyruvic acid	1.10	6.29E-06	2.95E-05	0.008	1.22E-05	0.298	0.002	0.086	8.43E-05
Oxoproline	1.20	8.40E-06	3.66E-05	0.036	4.47E-05	0.967	0.004	0.034	4.58E-05
Alpha-KG	1.26	1.09E-05	4.20E-05	4.24E-05	2.59E-05	0.096	0.877	0.006	0.003
G-6-P	1.24	1.10E-05	4.20E-05	0.0002	0.007	0.091	0.920	0.002	0.026
Palmitic acid	1.17	1.30E-05	4.67E-05	6.83E-06	0.002	0.0001	0.756	0.667	0.560
Glycolic acid	1.05	2.26E-05	7.65E-05	0.001	0.004	0.061	0.187	0.033	0.017
Fumaric acid	1.49	5.16E-05	0.0001	8.13E-06	0.219	0.942	0.006	0.0001	0.303
Glyceric acid	1.63	0.0001	0.0004	2.16E-05	0.731	0.058	0.039	0.001	0.620
Tryptophane	1.11	0.0002	0.0008	0.017	0.005	0.411	0.048	0.234	0.012
Stearic acid	1.02	0.0003	0.001	0.0001	0.0002	0.005	0.258	0.805	0.258
Leucine	1.09	0.0005	0.001	0.168	0.0006	0.057	0.008	0.779	0.009
Galactonic acid	1.16	0.001	0.002	5.86E-05	0.074	0.001	0.490	0.165	0.871
Capric acid	1.00	0.002	0.006	0.078	0.034	0.910	0.250	0.088	0.035
2,3-DHBA	1.22	0.004	0.011	0.019	0.056	0.707	0.914	0.0007	0.019
2-H-2MBA	1.01	0.005	0.011	0.002	0.018	0.127	0.428	0.046	0.249
1,5-anhydroglucitol	1.07	0.009	0.021	0.014	0.445	0.581	0.008	0.002	0.770
Succinic acid	1.01	0.011	0.022	0.0004	0.759	0.268	0.039	0.045	0.595
Lysine	1.17	0.011	0.022	0.658	0.175	0.027	0.072	0.016	0.126
Aspartic acid	1.0	0.017	0.035	0.001	0.010	0.024	0.932	0.925	0.983
Oleic acid	1.14	0.019	0.037	0.508	0.6996	0.0071	0.397	0.076	0.014
Glycerol	1.20	0.025	0.046	0.034	0.010	0.024	0.932	0.925	0.983
Malate	1.22	0.026	0.046	0.055	0.624	0.245	0.228	0.004	0.134
Serine	1.0	0.027	0.048	0.547	0.018	0.138	0.035	0.293	0.116

*ANOVA* Analysis of variance, *VIP* Variation importance in the projection, *FDR* False discovery rate, *H* Healthy, *PTC* Papillary thyroid cancer, *FTC* Folicular thyroid cancer, *MTC* Medullary thyroid cancer, *2-HB* 2-hyroxybutanoic acid, *3-HB* 3-hydroxybutyric acid, *Alpha-KG* Alpha-ketoglutaric acid, *G-6-P* Glucose-6-phosohate, *2,3-DHBA* 2,3-dihydroxybutanoic acid, *2-H-2MBA* 2-hydroxy-2methylbutanoic acid

^a^*p*-value < 0.05 is considered statistically significant based on the one-way ANOVA and Tukey’s post hoc test

### Feature importance and biomarker identification

**Fig. 3:**
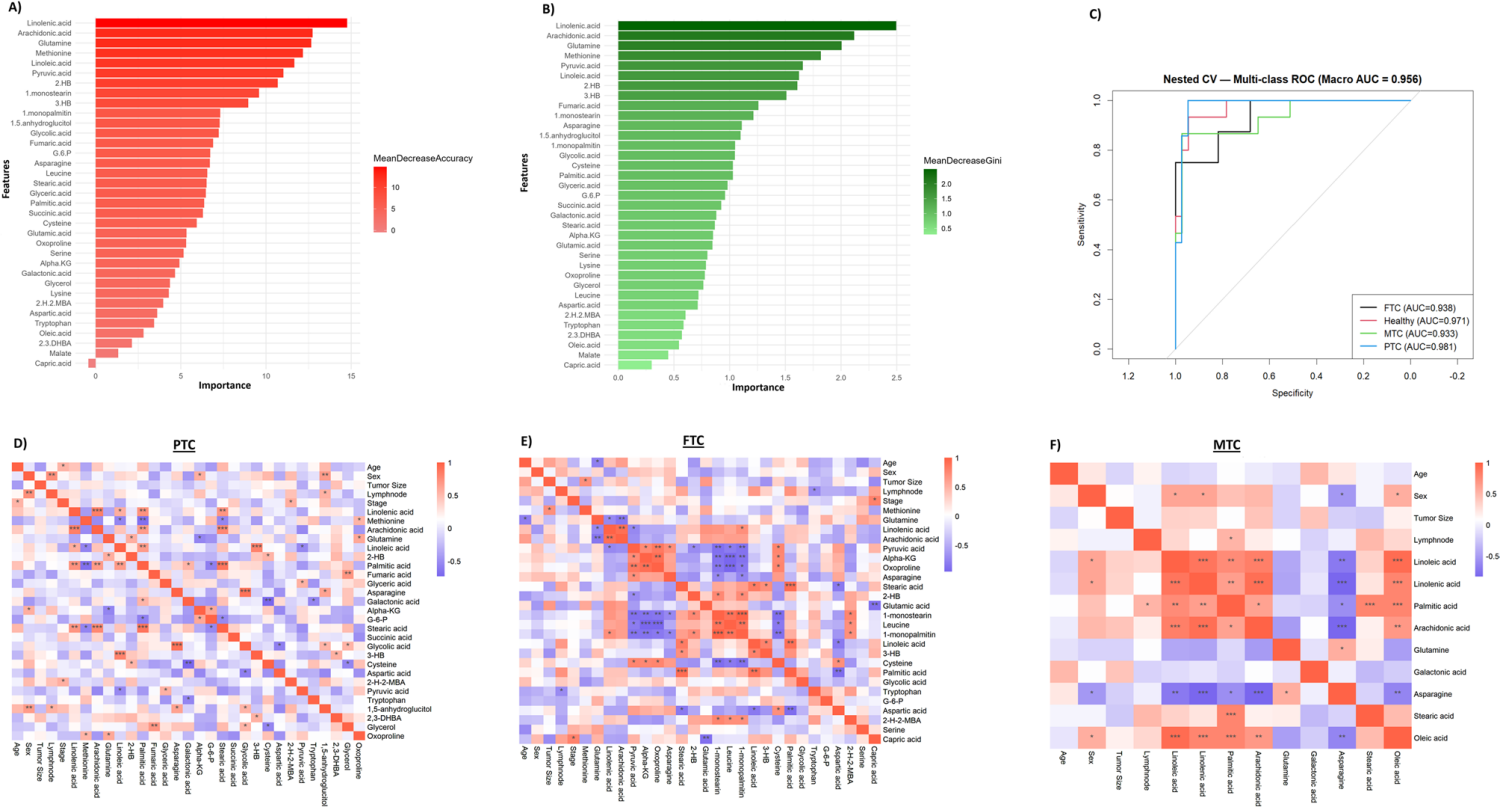
Integrated analysis of metabolite importance and correlation with clinical features in thyroid cancer subtypes.** A–B** Ranking of metabolite importance using Random Forest algorithms based on **A** Mean Decrease Accuracy and **B** Mean Decrease Gini, identifying key discriminatory features across sample groups. **C** Receiver Operating Characteristic (ROC) curves for multi-class classification of thyroid cancer subtypes and healthy controls, showing high diagnostic performance with a macro-AUC of 0.93. **D–F** Heatmaps depicting Pearson correlation coefficients between clinical variables and metabolite levels in **D** papillary thyroid carcinoma (PTC), **E** follicular thyroid carcinoma (FTC), and **F** medullary thyroid carcinoma (MTC). Colors indicate the strength and direction of the correlation (blue: negative, red: positive). Significant correlations are indicated by asterisks (*p < 0.05, **p < 0.01, ***p < 0.001)

An RF classifier was employed to uncover the most discriminative metabolites for distinguishing between thyroid cancer subtypes and healthy controls. Feature importance was evaluated using two robust metrics, including MDA and MDG. As shown in the ranked importance list (Fig. 3A), the top metabolite was linolenic acid, which yielded the highest importance score of MDA = 14.75, indicating a substantial impact on model performance. Other top-ranked features included: arachidonic acid (MDA = 12.72), glutamine (MDA = 12.65), methionine (MDA = 12.16), linoleic (MDA = 11.66). These metabolites are predominantly involved in lipid metabolism, inflammatory signaling, and amino acid turnover, which aligns with known metabolic reprogramming in cancer. MDG assesses feature contribution to node purity within decision trees (Fig. 3B). The MDG results reinforced the findings from MDA, with the same top features emerging. Again, linolenic acid and arachidonic acid ranked highest (Gini = 2.49 and 2.11, respectively), followed by glutamine (Gini = 2.00), methionine (Gini = 1.81), indicating a consistent pattern of metabolic relevance. The diagnostic capability of the RF model was evaluated using multi-class ROC analysis (Fig. 3C), which incorporated four groups: PTC, MTC, FTC, and healthy controls. The model achieved a macro-averaged AUC of 0.956, reflecting high overall accuracy. Class-specific AUCs were highest for PTC (AUC = 0.981), suggesting strong metabolic separability. The FTC and MTC presented relatively lower AUC values (0.938 and 0.933, respectively), indicating overlapping profiles with other subtypes, consistent with their known clinical complexity. The healthy control group was distinctly separated, affirming the ability of plasma metabolomics to capture malignant alterations.

### Correlation analysis

Heatmaps reveal correlations between clinical parameters, including age, tumor size, lymph node involvement, stage, and metabolite levels across different thyroid cancer subtypes. Within the PTC group (Fig. 3D), age showed weak to moderate correlations, which were significantly connected to disease stage (*p* < 0.05, *r* = 0.54). Although sex demonstrated limited correlations, it was linked considerably to 1,5-anhydroglycerol (*p* < 0.05, *r* = 0.63) and lymph node involvement (*p* < 0.01, *r* = 0.75). Tumor size presented a few significant metabolic associations, suggesting a minimal direct influence. Interactions among metabolites in this group exhibited strong correlations, particularly between linolenic acid and arachidonic acid, which showed a positive correlation (*p* < 0.001, *r* = 0.83). Additionally, palmitic acid showed a robust positive correlation with stearic acid (*p* < 0.001, *r* = 0.89), indicating a consistent metabolic relationship across the examined groups. In the FTC group (Fig. 3E), age showed weak to moderate correlations with metabolites, with a significant negative correlation with glutamine (*p* < 0.05, *r* = −0.78). Tumor size showed a limited, but significant, positive correlation with methionine (*p* < 0.05, *r* = 0.72). Lymph node involvement demonstrated a notable negative correlation with tryptophan (*p* < 0.05, *r* = −0.71). Disease stage also correlated positively and significantly with capric acid (*p* < 0.05, *r* = 0.78). Among the metabolites, glutamine exhibited significant negative correlations with arachidonic and linolenic acids (*p* < 0.01, *r* = −0.83; *p* < 0.05, *r* = −0.83, respectively), implying possible metabolic interactions related to inflammation or oxidative stress. Linoleic acid illustrated strong internal metabolic relationships, positively correlating with palmitic acid (*p* < 0.01, *r* = 0.91) and 3-HB (*p* < 0.05, *r* = 0.79), while negatively correlating with aspartic acid (*p* < 0.05, *r* = −0.82). Moreover, palmitic and stearic acids displayed a strong positive inter-correlation (*p* < 0.001, *r* = 0.93), suggesting coordinated metabolic activity. In the MTC group (Fig. 3F), age and tumor size primarily showed weak correlations, lacking significant metabolic links. Lymph node involvement revealed a significant positive correlation with palmitic acid (*p* < 0.05, *r* = 0.51), supporting the notion of metabolic shifts related to disease progression. Linoleic and linolenic acids exhibited strong positive correlations with arachidonic acid (*p* < 0.001, *r* = 0.82 and *r* = 0.91, respectively) and palmitic acid, highlighting the interconnected metabolic pathways within this group. Moreover, asparagine presented a substantial negative correlation with arachidonic and linolenic acid (*p* < 0.001, *r* = −0.82 and *r* = −0.83, respectively).

### Metabolic pathway analysis

**Fig. 4:**
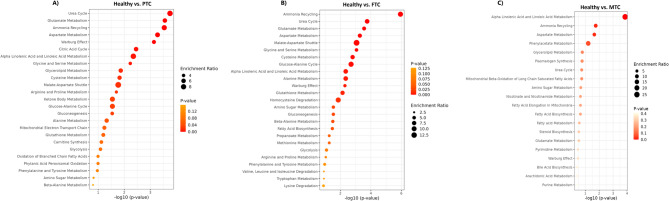
Pathway enrichment analysis comparing healthy controls with thyroid cancer subtypes, including PTC, FTC, and MTC **A**, **B**, **C**. Dot plots show significantly enriched metabolic pathways, where the x-axis represents statistical significance (–log10 *p*-value), the dot size indicates the enrichment ratio, and the dot color corresponds to the *p*-value

To calculate the class enrichment, metabolic set enrichment analysis was performed using the Small Molecule Pathway Database (SMPDB) (Fig. 4A-C). Pathway enrichment analysis revealed distinct metabolic alterations between healthy controls and thyroid cancer subtypes. In PTC, broad dysregulation was observed, particularly in the urea cycle, glutamate and aspartate metabolism, amino acid pathways, and central carbon metabolism, reflecting a global metabolic reprogramming. In FTC, significant enrichment was also detected in ammonia recycling, urea cycle, and multiple amino acid pathways, along with alterations in fatty acid biosynthesis and glucose metabolism, indicating combined amino acid and lipid remodeling. In contrast, MTC exhibited fewer but more selective changes, primarily affecting lipid metabolism (linoleic/linolenic acid, glycerolipid, and steroid biosynthesis) and specific amino acid pathways. these findings highlight that although PTC, FTC, and MTC share some common metabolic disturbances (ammonia recycling, amino acid metabolism), each cancer subtype exhibits a distinct metabolic fingerprint. PTC exhibits broader dysregulation across central carbon and amino acid metabolism, whereas FTC involves both amino acid and lipid metabolic remodeling, and MTC appears to be more restricted, focusing on lipid metabolism and selected amino acid pathways. All of the enriched metabolic pathways can be found in Tables S1-S3.

## Discussion

Currently, the preoperative assessment of thyroid nodules primarily depends on cytology through FNA, often resulting in indeterminate findings for a notable portion of cases​ [7]. Hence, there is an urgent need for non-invasive biomarkers that can differentiate between malignant and benign nodules, as well as between aggressive and indolent forms of the disease. Metabolomics offers a valuable tool for identifying potential biomarkers [6]. By examining the metabolomic profiles of thyroid carcinomas, we can identify metabolic disturbances that are specific to certain subtypes. This approach enhances our understanding of their biological foundations and aids in discovering biomarkers that improve classification and enable targeted treatments. The GC/MS-based metabolomics analysis indicated that thyroid carcinomas undergo significant metabolic alteration compared to healthy subjects. Each subtype, including PTC, FTC, and MTC, exhibited distinct variations in metabolic profiles, particularly regarding lipid and amino acid metabolism. Several circulating metabolites were identified as significantly altered and highly effective in distinguishing between the presence and absence of cancer, underscoring critical metabolic pathways exploited by these tumors.

A notable and consistent finding across all subtypes was the dysregulation of lipid metabolism, specifically the depletion of circulating polyunsaturated fatty acids (PUFAs). Plasma levels of linoleic acid (18:2, omega-6), linolenic acid (18:3, omega-3), and arachidonic acid (20:4, omega-6) were significantly lower in patients with thyroid cancers. This trend likely indicates increased uptake and metabolic conversion of these fatty acids by tumors. PUFAs serve not only as structural elements in cellular membranes but also as precursors for bioactive lipid mediators [15, 16]. Linoleic and linolenic acids are enzymatically desaturated and elongated to form arachidonic acid, which is then metabolized by cyclooxygenase (COX) and lipoxygenase (LOX) enzymes to generate eicosanoids, such as prostaglandin E2 (PGE2), leukotrienes, and thromboxanes [17, 18]. These lipid mediators promote inflammation, angiogenesis, and tumor growth, establishing a biochemical link between plasma lipid depletion and tumor-driven lipid signaling [19]. Notably, the elevated expression of COX-2 in PTC underlines this mechanism, indicating enhanced conversion of arachidonic acid to PGE2 in the tumor microenvironment [20]. Prior metabolomics studies support these findings, reporting lower PUFA levels in thyroid cancer and patient biofluids [1, 21]. Furthermore, machine learning analysis has highlighted their diagnostic importance, underscoring their pivotal roles as membrane constituents and signaling precursors. In contrast to the depletion of free polyunsaturated FAs, we observed aberrations in glyceride metabolism exemplified by 1-monostearin (1-stearoylglycerol). This monoacylglycerol was present at the lowest levels in FTC patients and at intermediate levels in PTC and MTC patients. Monostearin is a breakdown product of triglycerides, and its variable levels suggest differences in lipolytic activity or lipid storage among subtypes [22]. Low plasma monostearin in FTC patients could indicate more complete uptake or utilization of circulating triglyceride-derived fatty acids by FTC tumors. Although monostearin is less commonly discussed in the literature, similar monoacylglycerols have been reported as metabolic markers in other cancers, including prostate cancer risk, which is inversely associated with circulating 1-monostearoylglycerol [23]. Our findings suggest that the FTC relies heavily on circulating lipids for energy or as a source of biomass. Another compelling marker of lipid metabolism reprogramming in thyroid cancer is the elevated plasma level of 3-hydroxybutyrate (3-HB), a ketone body produced from acetyl-CoA during the β-oxidation of fatty acids. This increase signifies a shift toward systemic fat utilization and possibly a mild ketogenic state. Consistent with our results, other studies have identified elevated 3-HB in thyroid cancer patients [12, 13, 24, 25], linking it to altered lipolysis and energy reallocation. While the Warburg effect (aerobic glycolysis) dominates cancer metabolism [26], recent research suggests many cancers exhibit metabolic flexibility, including the ability to oxidize ketones or fatty acids when needed​ [27]. Alternatively, the elevated ketones may essentially be a host effect. In particular, thyroid cancer patients with a significant tumor burden might experience mild cachexia or intermittent fasting states, leading to hepatic ketogenesis [28]. Regardless, the high circulating levels of 3-HB and pyruvate in our patients indicate a dual aspect of tumor energy reprogramming: enhanced glycolysis (overflow of pyruvate) and enhanced fat oxidation (overflow of 3-HB). Indeed, we found pyruvate levels were highest in FTC, suggesting robust glycolytic flux in this subtype. Even in PTC, a study noted that pyruvate and TCA intermediates were elevated, consistent with an upregulated TCA cycle ​ [29]. This may seem paradoxical given the classic Warburg effect, which implies reliance on glycolysis with lactate excretion rather than complete TCA oxidation. However, our findings and those of others [30] indicate that thyroid tumors, especially FTC, can exhibit a Warburg-like high glycolytic rate and active mitochondrial oxidation simultaneously. FTC’s metabolic profile of high pyruvate and active TCA plus glutaminolysis fits a pattern of a tumor aggressively fueling the TCA cycle from multiple inputs like glucose via pyruvate, glutamine via α-ketoglutarate, and possibly fatty acids/ketones. This could confer a growth advantage and might relate to why FTC can show more propensity for distant metastasis than PTC.

In addition to lipid alterations, our metabolomics profiling revealed extensive reprogramming of amino acid metabolism across all thyroid cancer subtypes. Plasma levels of several amino acids and their derivatives were significantly disrupted, reflecting the tumor’s metabolic demands for anabolic growth and signaling. Among the most significantly impacted were methionine and glutamine, which were also confirmed by machine learning analysis, having emerged as key metabolic nodes with subtype-specific alterations and substantial diagnostic value. Methionine, an essential amino acid required for protein synthesis, methylation reactions via S-adenosylmethionine, and polyamine biosynthesis [30], was markedly altered in thyroid cancer patients. It is biologically significant that tumors are often “methionine-addicted”, relying on exogenous methionine for proliferation. This dependency, known as the Hoffman effect, arises because cancer cells lack the ability to recycle methionine efficiently [31]. In our study, the increase of methionine in PTC and FTC was more prominent than in MTC. A similar pattern of methionine elevation was obtained in two separate studies, which showed that cysteine and methionine metabolism are upregulated in PTC patients [29, 32]. Strikingly, we found that 2-HB levels were elevated in PTC and FTC patients. 2-HB is a byproduct of altered amino acid and glutathione metabolism. Elevated 2-HB is often associated with increased flux through the methionine/homocysteine trans-sulfuration pathway, which produces cysteine for glutathione synthesis while yielding 2-ketobutyrate and is quickly reduced to 2-HB ​ [33]. In cancer, high oxidative stress can drive trans-sulfuration as cells attempt to bolster glutathione levels for redox defense [34]. This is supported by the concurrent increase in oxoproline (pyroglutamate) observed especially in FTC patients. Oxoproline is an intermediate of the γ-glutamyl cycle in glutathione turnover; accumulation of oxoproline signifies glutathione depletion or rapid cycling [35]. Notably, oxoproline has been found to be more abundant in tumor tissues in nasopharyngeal carcinoma [36] and bladder cancer [37]. In our FTC cases, high oxoproline together with 2-HB strongly suggests that FTC tumors experience significant oxidative stress, prompting an augmented antioxidant response. This linkage between methionine cycle upregulation, 2-HB, and glutathione metabolism exemplifies how disparate metabolite changes can be interconnected: here via one-carbon metabolism feeding into redox homeostasis. Another facet of redox and metabolic stress is tryptophan depletion, which was observed in our MTC patients while remaining relatively stable in PTC and FTC. Tryptophan is an essential amino acid and a precursor for serotonin and kynurenine [38]. In cancer, the indoleamine 2,3-dioxygenase (IDO) pathway is frequently activated in immune and tumor cells, converting tryptophan into kynurenine [39]. This not only helps cancer cells manage oxidative stress but also induces immune tolerance [40]. MTC tumors or their microenvironment may upregulate IDO, leading to tryptophan consumption and accumulation of immunosuppressive kynurenine. In thyroid cancer, IDO1 overexpression has indeed been noted and correlates with suppressed immune cell activity ​ [41]. Our data suggest that MTC, in particular, may exploit this pathway, as evidenced by the low systemic tryptophan levels, which serve as a metabolic adaptation.

One of the most notable alterations in our analysis was in the glutamine–glutamate axis, which differed by subtype, as highlighted by our machine learning analysis. Although context-dependent, we observed notable fluctuations in glutamine levels across different subtypes, aligning with its established role in cancer as both a nitrogen donor for nucleotide and amino acid production and as a carbon source through its conversion to glutamate and subsequently to α-ketoglutarate, which supports the TCA cycle [42]. Interestingly, our findings indicated distinct subtype-specific patterns in glutamine metabolism. FTC cases showed the most significant increase in plasma glutamine levels, while PTC displayed moderate rises, and MTC exhibited variable but often elevated levels. This variability could represent the interplay between tumor glutamine utilization and muscle proteolysis in the patients, affecting overall glutamine availability. In accordance with the present results, previous studies have demonstrated that glutamine levels increased in plasma samples of patients with thyroid cancer [1, 32, 43].

Our findings not only align with existing plasma-based metabolomics studies in PTC and MTC but also offer novel insights into the distinct metabolic behavior of FTC, an area that has remained underexplored in plasma-focused analyses, underscoring the metabolic heterogeneity across thyroid cancer subtypes. While the consistency with prior serum/plasma-based studies adds robustness to our data, it is important to note that some of those reports aggregated FTC with PTC under the broader DTC category, potentially overlooking subtype-specific signatures. Our study, although limited by its cohort size and reliance on plasma profiling, helps fill this gap by emphasizing the unique metabolic fingerprint of FTC.

## Conclusion

These findings suggest that integrating metabolomics with advanced machine learning techniques effectively differentiates between various types of thyroid cancer and healthy individuals. Identifying critical metabolic markers, such as PUFAs, not only aids in classification but also enables biological interpretation and potential therapeutic targeting. Lowering these lipids in circulation among all cancer patients, along with their recognized oncogenic roles when modified in tumors, supports the notion that thyroid cancers utilize systemic lipid resources for both growth and signaling. Furthermore, alterations in amino acid metabolism, particularly those related to glutamine and methionine, highlight the metabolic reprogramming characteristic of thyroid malignancies. The variations observed between PTC, FTC, and MTC underscore the influence of the tumor cell-of-origin and dominant oncogenic drivers on metabolic phenotypes. Despite these promising results, it is essential to acknowledge the limited sample size in this study, particularly for the FTC groups, which may impact the generalizability and strength of the findings. Future research should prioritize larger and more balanced cohorts to validate these outcomes and further investigate subtype-specific metabolic signatures. Additionally, increasing sample diversity and incorporating multi-omics data could significantly enhance the diagnostic and therapeutic applications of metabolomics in thyroid disorders.

## Supplementary Information

Supplementary Material 1.

## Data Availability

All data generated or analyzed during this study are included in this article and its supplementary information files.

## Abbreviations

PTC: Papillary thyroid cancer
FTC: Follicular thyroid cancer
MTC: Medullary thyroid cancer
TCA: Tricarboxylic acid cycle
EDTA: Ethylenediaminetetraacetic acid
TMS: Trimethylsilylation
QC: Quality control
GC/MS: Gas chromatography–quadrupole mass spectrometry
PQN: Probabilistic quotient normalization
PCA: Principal component analysis
PLS-DA: Partial least squares discriminant analysis
OPLS-DA: Orthogonal partial least squares discriminant analysis
CV-ANOVA: Cross-validated predictive residuals
ANOVA: Analysis of variance
VIP: Variation importance in the projection
FDR: False discovery rate
RF: Random forest
MDA: Mean decrease accuracy
MDG: Mean decrease gini
SMPDB: Small molecule pathway database
2-HB: 2-hyroxybutanoic acid
3-HB: 3-hydroxybutyric acid
Alpha-KG: Alpha-ketoglutaric acid
G-6-P: Glucose-6-phosohate
2,3-DHBA: 2,3-dihydroxybutanoic acid
2-H-2MBA: 2-hydroxy-2methylbutanoic acid
PUFAs: Polyunsaturated fatty acids
COX: Cyclooxygenase
LOX: Lipoxygenase
