# Supplementary material for: Metabolomics fingerprinting of thyroid malignancies: a GC/MS-based approach for subtype classification and biomarker discovery
Source: BMC Cancer. 2025 Oct 15;25:1586. doi: 10.1186/s12885-025-15073-0 (PMC12523154; doi:10.1186/s12885-025-15073-0)
Supplement: Supplementary file 1 — Supplementary Material 1. [file 12885_2025_15073_MOESM1_ESM.docx]

**Metabolomics Fingerprinting of Thyroid Malignancies: A GC/MS-Based Approach for Subtype Classification and Biomarker Discovery**

Raziyeh Abooshahab^1,2,3^, Maryam Zarkesh^1^, Mehdi Hedayati^1*^

^1^Cellular and Molecular Endocrine Research Center, Research Institute for Endocrine Molecular Biology, Research Institute for Endocrine Sciences, Shahid Beheshti University of Medical Sciences, Tehran, Iran

^2^Curtin Medical School, Curtin University, Bentley 6102, Australia

^3^Curtin Health Innovation Research Institute, Bentley 6102, Australia

**Correspondence:**

Mehdi Hedayati (Ph.D.), Professor in Biochemistry

Cellular and Molecular Endocrine Research Center (CMERC), Research Institute for Endocrine Sciences of Shahid Beheshti University of Medical Sciences, Tehran, Iran

**PO** Box: 19395-4763

**Tel:** +98(21)22432500

**Fax:** +98(21)22416264

**Email:** [**hedayati@endocrine.ac.ir**](mailto:hedayati@endocrine.ac.ir) **&** [**hedayati47@gmail.com**](mailto:hedayati47@gmail.com)

**Supplemental Figure S1:** Score plot of Principal Component Analysis (PCA).

**Supplemental Table S1:** Pathway Enrichment analysis of altered metabolites between Healthy and PTC

**Supplemental Table S2:** Pathway Enrichment analysis of altered metabolites between Healthy and FTC

**Supplemental Table S3:** Pathway Enrichment analysis of altered metabolites between Healthy and MTC


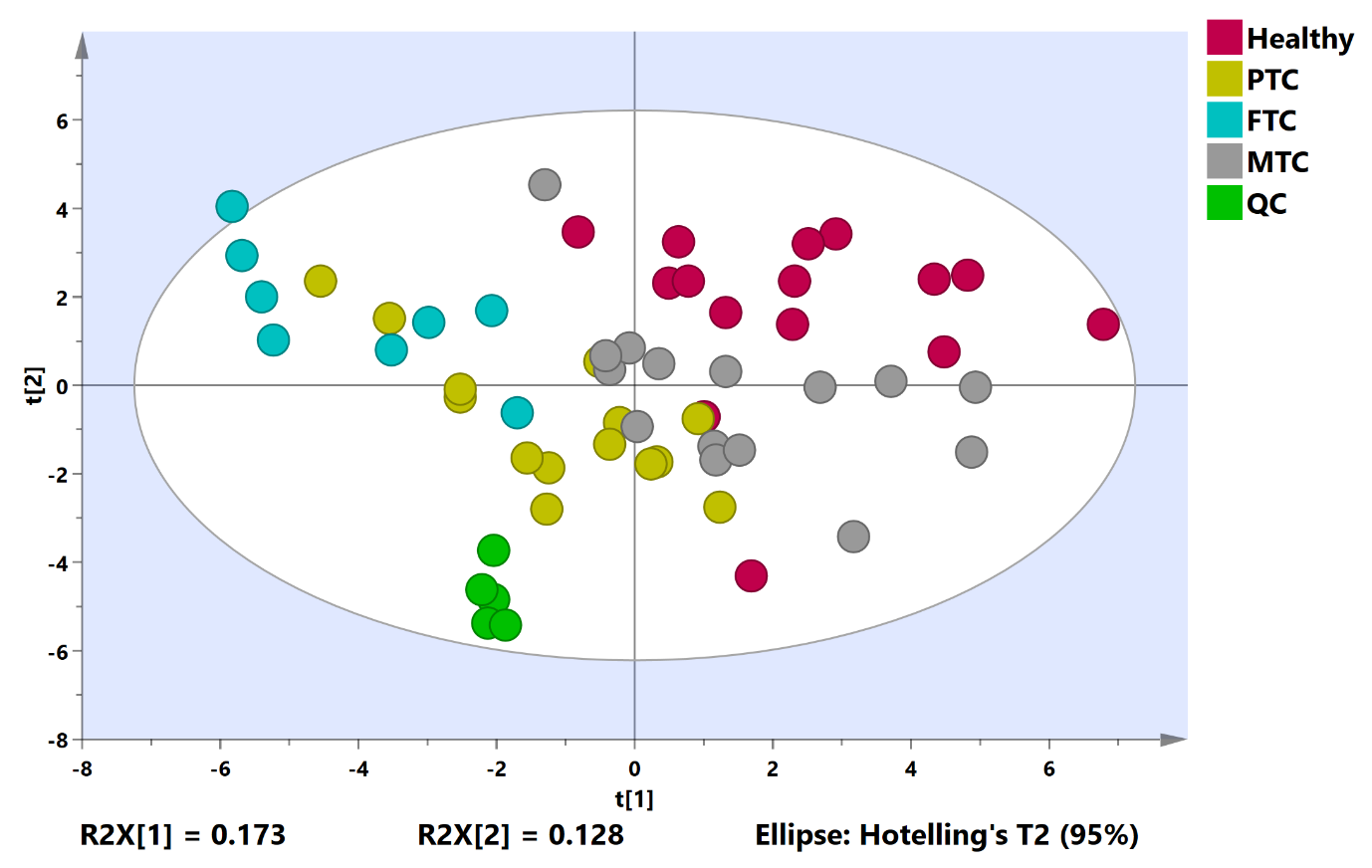


**Figure S1:** **Score plot of Principal Component Analysis (PCA) showing the distribution of samples based on the first two principal components (PC1 and PC2).** Each point represents an individual sample. The color coding corresponds to the following groups: Healthy (dark pink), Papillary Thyroid Carcinoma (PTC, yellow), Follicular Thyroid Carcinoma (FTC, cyan), Medullary Thyroid Carcinoma (MTC, gray), and Quality Control (QC, green**)**. The model explains 17.3% and 12.8% of the variance in the first and second components, respectively.

**Table S1:** Pathway Enrichment analysis of altered metabolites between Healthy and PTC

| **Metabolic Pathways** | **^a^Total Cmpd** | **^b^Hits** | **^c^Raw p** | **^d^FDR** |
| --- | --- | --- | --- | --- |
| Urea Cycle | 28 | 5 | 0.000176 | 0.00953 |
| Glutamate Metabolism | 48 | 6 | 0.000277 | 0.00953 |
| Ammonia Recycling | 31 | 5 | 0.000292 | 0.00953 |
| Aspartate Metabolism | 35 | 5 | 0.000528 | 0.0129 |
| Warburg Effect | 57 | 6 | 0.000726 | 0.0142 |
| Citric Acid Cycle | 32 | 4 | 0.00351 | 0.0573 |
| Alpha Linolenic Acid and Linoleic Acid Metabolism | 17 | 3 | 0.00447 | 0.0626 |
| Glycine and Serine Metabolism | 59 | 5 | 0.00586 | 0.0718 |
| Glycerolipid Metabolism | 25 | 3 | 0.0136 | 0.148 |
| Cysteine Metabolism | 26 | 3 | 0.0151 | 0.148 |
| Malate-Aspartate Shuttle | 10 | 2 | 0.017 | 0.152 |
| Arginine and Proline Metabolism | 52 | 4 | 0.02 | 0.164 |
| Ketone Body Metabolism | 13 | 2 | 0.0284 | 0.189 |
| Glucose-Alanine Cycle | 13 | 2 | 0.0284 | 0.189 |
| Gluconeogenesis | 33 | 3 | 0.0289 | 0.189 |
| Alanine Metabolism | 17 | 2 | 0.0471 | 0.288 |
| Mitochondrial Electron Transport Chain | 19 | 2 | 0.0577 | 0.333 |
| Glutathione Metabolism | 20 | 2 | 0.0634 | 0.345 |
| Carnitine Synthesis | 22 | 2 | 0.0751 | 0.387 |
| Glycolysis | 23 | 2 | 0.0813 | 0.398 |
| Oxidation of Branched Chain Fatty Acids | 26 | 2 | 0.101 | 0.448 |
| Phytanic Acid Peroxisomal Oxidation | 26 | 2 | 0.101 | 0.448 |
| Phenylalanine and Tyrosine Metabolism | 27 | 2 | 0.107 | 0.457 |
| Amino Sugar Metabolism | 33 | 2 | 0.15 | 0.589 |
| Beta-Alanine Metabolism | 34 | 2 | 0.157 | 0.589 |
| Fatty Acid Biosynthesis | 35 | 2 | 0.165 | 0.589 |
| Phenylacetate Metabolism | 9 | 1 | 0.174 | 0.589 |
| Homocysteine Degradation | 9 | 1 | 0.174 | 0.589 |
| Tyrosine Metabolism | 70 | 3 | 0.176 | 0.589 |
| Galactose Metabolism | 38 | 2 | 0.187 | 0.589 |
| Pyruvaldehyde Degradation | 10 | 1 | 0.192 | 0.589 |
| Purine Metabolism | 73 | 3 | 0.192 | 0.589 |
| Propanoate Metabolism | 42 | 2 | 0.219 | 0.63 |
| Methionine Metabolism | 42 | 2 | 0.219 | 0.63 |
| Taurine and Hypotaurine Metabolism | 12 | 1 | 0.226 | 0.632 |
| Spermidine and Spermine Biosynthesis | 18 | 1 | 0.319 | 0.845 |
| Butyrate Metabolism | 19 | 1 | 0.334 | 0.845 |
| Nucleotide Sugars Metabolism | 20 | 1 | 0.348 | 0.845 |
| Valine, Leucine and Isoleucine Degradation | 59 | 2 | 0.354 | 0.845 |
| Tryptophan Metabolism | 59 | 2 | 0.354 | 0.845 |
| Pantothenate and CoA Biosynthesis | 21 | 1 | 0.362 | 0.845 |
| Betaine Metabolism | 21 | 1 | 0.362 | 0.845 |
| Transfer of Acetyl Groups into Mitochondria | 22 | 1 | 0.376 | 0.856 |
| Inositol Phosphate Metabolism | 24 | 1 | 0.402 | 0.896 |
| Plasmalogen Synthesis | 26 | 1 | 0.428 | 0.931 |
| Mitochondrial Beta-Oxidation of Long Chain Saturated Fatty Acids | 28 | 1 | 0.452 | 0.951 |
| Pentose Phosphate Pathway | 29 | 1 | 0.464 | 0.951 |
| Inositol Metabolism | 30 | 1 | 0.475 | 0.951 |
| Lysine Degradation | 30 | 1 | 0.475 | 0.951 |
| Starch and Sucrose Metabolism | 31 | 1 | 0.487 | 0.954 |
| Nicotinate and Nicotinamide Metabolism | 35 | 1 | 0.53 | 0.998 |
| Fatty Acid Elongation In Mitochondria | 35 | 1 | 0.53 | 0.998 |
| Fatty acid Metabolism | 43 | 1 | 0.606 | 1 |
| Pyruvate Metabolism | 47 | 1 | 0.639 | 1 |
| Steroid Biosynthesis | 48 | 1 | 0.647 | 1 |
| Pyrimidine Metabolism | 57 | 1 | 0.711 | 1 |
| Bile Acid Biosynthesis | 65 | 1 | 0.759 | 1 |
| Arachidonic Acid Metabolism | 67 | 1 | 0.77 | 1 |

^a^ Total Cmpd: total number of compounds in the pathway,  ^b^ Hit: actually matched number from the data

^c^ Raw p: p-value calculated from the enrichment analysis, ^d^ FDR: p-value adjusted using False Discovery Rate

**Table S2:** Pathway Enrichment analysis of altered metabolites between Healthy and FTC

| **Metabolic Pathways** | **^a^Total Cmpd** | **^b^Hits** | **^c^Raw p** | **^d^FDR** |
| --- | --- | --- | --- | --- |
| Ammonia Recycling | 31 | 7 | 1.15E-06 | 0.000113 |
| Urea Cycle | 28 | 5 | 0.000176 | 0.00861 |
| Glutamate Metabolism | 48 | 6 | 0.000277 | 0.00904 |
| Aspartate Metabolism | 35 | 5 | 0.000528 | 0.0129 |
| Malate-Aspartate Shuttle | 10 | 3 | 0.000868 | 0.0143 |
| Glycine and Serine Metabolism | 59 | 6 | 0.000877 | 0.0143 |
| Cysteine Metabolism | 26 | 4 | 0.00158 | 0.0222 |
| Glucose-Alanine Cycle | 13 | 3 | 0.00199 | 0.0243 |
| Alpha Linolenic Acid and Linoleic Acid Metabolism | 17 | 3 | 0.00447 | 0.0438 |
| Alanine Metabolism | 17 | 3 | 0.00447 | 0.0438 |
| Warburg Effect | 57 | 5 | 0.00504 | 0.0449 |
| Glutathione Metabolism | 20 | 3 | 0.0072 | 0.0588 |
| Homocysteine Degradation | 9 | 2 | 0.0138 | 0.104 |
| Amino Sugar Metabolism | 33 | 3 | 0.0289 | 0.189 |
| Gluconeogenesis | 33 | 3 | 0.0289 | 0.189 |
| Beta-Alanine Metabolism | 34 | 3 | 0.0313 | 0.191 |
| Fatty Acid Biosynthesis | 35 | 3 | 0.0337 | 0.194 |
| Propanoate Metabolism | 42 | 3 | 0.0538 | 0.278 |
| Methionine Metabolism | 42 | 3 | 0.0538 | 0.278 |
| Glycolysis | 23 | 2 | 0.0813 | 0.398 |
| Arginine and Proline Metabolism | 52 | 3 | 0.0906 | 0.423 |
| Phenylalanine and Tyrosine Metabolism | 27 | 2 | 0.107 | 0.478 |
| Valine, Leucine and Isoleucine Degradation | 59 | 3 | 0.121 | 0.495 |
| Tryptophan Metabolism | 59 | 3 | 0.121 | 0.495 |
| Lysine Degradation | 30 | 2 | 0.128 | 0.502 |
| Citric Acid Cycle | 32 | 2 | 0.142 | 0.537 |
| Nicotinate and Nicotinamide Metabolism | 35 | 2 | 0.165 | 0.596 |
| Phenylacetate Metabolism | 9 | 1 | 0.174 | 0.596 |
| Tyrosine Metabolism | 70 | 3 | 0.176 | 0.596 |
| Pyruvaldehyde Degradation | 10 | 1 | 0.192 | 0.608 |
| Purine Metabolism | 73 | 3 | 0.192 | 0.608 |
| Taurine and Hypotaurine Metabolism | 12 | 1 | 0.226 | 0.67 |
| Phosphatidylethanolamine Biosynthesis | 12 | 1 | 0.226 | 0.67 |
| Ketone Body Metabolism | 13 | 1 | 0.242 | 0.697 |
| Beta Oxidation of Very Long Chain Fatty Acids | 17 | 1 | 0.304 | 0.852 |
| Spermidine and Spermine Biosynthesis | 18 | 1 | 0.319 | 0.869 |
| Nucleotide Sugars Metabolism | 20 | 1 | 0.348 | 0.891 |
| Pantothenate and CoA Biosynthesis | 21 | 1 | 0.362 | 0.891 |
| Betaine Metabolism | 21 | 1 | 0.362 | 0.891 |
| Carnitine Synthesis | 22 | 1 | 0.376 | 0.891 |
| Transfer of Acetyl Groups into Mitochondria | 22 | 1 | 0.376 | 0.891 |
| Inositol Phosphate Metabolism | 24 | 1 | 0.402 | 0.891 |
| Glycerolipid Metabolism | 25 | 1 | 0.415 | 0.891 |
| Arachidonic Acid Metabolism | 67 | 2 | 0.416 | 0.891 |
| Oxidation of Branched Chain Fatty Acids | 26 | 1 | 0.428 | 0.891 |
| Phytanic Acid Peroxisomal Oxidation | 26 | 1 | 0.428 | 0.891 |
| Plasmalogen Synthesis | 26 | 1 | 0.428 | 0.891 |
| Selenoamino Acid Metabolism | 27 | 1 | 0.44 | 0.891 |
| Mitochondrial Beta-Oxidation of Long Chain Saturated Fatty Acids | 28 | 1 | 0.452 | 0.891 |
| Pentose Phosphate Pathway | 29 | 1 | 0.464 | 0.891 |
| Folate Metabolism | 29 | 1 | 0.464 | 0.891 |
| Inositol Metabolism | 30 | 1 | 0.475 | 0.896 |
| Starch and Sucrose Metabolism | 31 | 1 | 0.487 | 0.9 |
| Fatty Acid Elongation In Mitochondria | 35 | 1 | 0.53 | 0.961 |
| Galactose Metabolism | 38 | 1 | 0.56 | 0.997 |
| Sphingolipid Metabolism | 40 | 1 | 0.579 | 1 |
| Histidine Metabolism | 42 | 1 | 0.597 | 1 |
| Fatty acid Metabolism | 43 | 1 | 0.606 | 1 |
| Pyruvate Metabolism | 47 | 1 | 0.639 | 1 |
| Steroid Biosynthesis | 48 | 1 | 0.647 | 1 |
| Pyrimidine Metabolism | 57 | 1 | 0.711 | 1 |
| Bile Acid Biosynthesis | 65 | 1 | 0.759 | 1 |

^a^ Total Cmpd: total number of compounds in the pathway,  ^b^ Hit: actually matched number from the data

^c^ Raw p: p-value calculated from the enrichment analysis, ^d^ FDR: p-value adjusted using False Discovery Rate

**Table S3:** Pathway Enrichment analysis of altered metabolites between Healthy and MTC

| **Metabolic Pathways** | **^a^Total Cmpd** | **^b^Hits** | **^c^Raw p** | **^d^FDR** |
| --- | --- | --- | --- | --- |
| Alpha Linolenic Acid and Linoleic Acid Metabolism | 17 | 3 | 0.000136 | 0.0134 |
| Ammonia Recycling | 31 | 2 | 0.0177 | 0.729 |
| Aspartate Metabolism | 35 | 2 | 0.0223 | 0.729 |
| Phenylacetate Metabolism | 9 | 1 | 0.0614 | 1 |
| Glycerolipid Metabolism | 25 | 1 | 0.163 | 1 |
| Plasmalogen Synthesis | 26 | 1 | 0.169 | 1 |
| Urea Cycle | 28 | 1 | 0.18 | 1 |
| Mitochondrial Beta-Oxidation of Long Chain Saturated Fatty Acids | 28 | 1 | 0.18 | 1 |
| Amino Sugar Metabolism | 33 | 1 | 0.21 | 1 |
| Nicotinate and Nicotinamide Metabolism | 35 | 1 | 0.221 | 1 |
| Fatty Acid Elongation In Mitochondria | 35 | 1 | 0.221 | 1 |
| Fatty Acid Biosynthesis | 35 | 1 | 0.221 | 1 |
| Fatty acid Metabolism | 43 | 1 | 0.265 | 1 |
| Steroid Biosynthesis | 48 | 1 | 0.292 | 1 |
| Glutamate Metabolism | 48 | 1 | 0.292 | 1 |
| Pyrimidine Metabolism | 57 | 1 | 0.337 | 1 |
| Warburg Effect | 57 | 1 | 0.337 | 1 |
| Bile Acid Biosynthesis | 65 | 1 | 0.376 | 1 |
| Arachidonic Acid Metabolism | 67 | 1 | 0.385 | 1 |
| Purine Metabolism | 73 | 1 | 0.412 | 1 |

^a^ Total Cmpd: total number of compounds in the pathway,  ^b^ Hit: actually matched number from the data

^c^ Raw p: p-value calculated from the enrichment analysis, ^d^ FDR: p-value adjusted using False Discovery Rate
